# Intraocular osseous metaplasia presenting as a solid mass in chronic retinal detachment: a case report

**DOI:** 10.1186/s40942-021-00331-7

**Published:** 2021-10-13

**Authors:** Yi-Ran Chiou, Lei-Chi Wang, Yu-Bai Chou

**Affiliations:** 1 Department of Ophthalmology, Taipei Veterans General Hospital, No. 201, Section 2, Shi-Pai Road, Taipei, 11217 Taiwan; 2 Department of Pathology and Laboratory Medicine, Taipei Veterans General Hospital, Taipei, Taiwan; 3 School of Medicine, National Yang Ming Chiao Tung University, Taipei, Taiwan

**Keywords:** Cellular transformation, Chronic inflammation, Coat’s disease, Osseous metaplasia, Proliferative vitreoretinopathy, Retinal detachment

## Abstract

**Background:**

Intraocular osseous metaplasia is a rare histological finding associated with benign cellular transformation. Its development requires inflammatory cytokines and the process takes many years. Previous case reports of intraocular ossification manifested as linear calcification or white plaques. In contrast, our case presented with a tumor-like solid mass, in which a long-standing chronic inflammatory stimulation may contribute to the stunning appearance.

**Case presentation:**

This is a 48-year-old woman with past history of advanced Coat’s-like retinopathy and chronic retinal detachment in the left eye for 12 years. She underwent vitreoretinal surgery to prevent phthisis bulbi. During the operation, a 9 mm solid mass was found embedded within the proliferative tissue above the retina and was removed. Pathological findings revealed bone formation in the center of the mass surrounded by fibrous metaplasia and focal gliotic changes. Layers of cohesive cells were found lining on the external side of the mass, and further immuno-histochemical study suggested them retinal pigment epithelial cells. Postoperatively, the retina was attached with stable visual acuity and normal intraocular pressure.

**Conclusion:**

To our knowledge, the appearance of a tumor-like mass representing intraocular osseous metaplasia in eyes with chronic inflammation or retinal detachment has not been reported in previous case reports. This case emphasizes the importance of considering osseous metaplasia as one of the differential diagnoses of an unknown intraocular mass, especially in eyes with great severity of chronic inflammation. Also, our immuno-histochemical study provided more evidence on the pathological role of retinal pigment epithelial cells in developing ossification.

## Background

Osseous metaplasia from retinal tissues is rare, previously described in case reports or case series [1–3]. It was mostly found in enucleated eyes, in which a long-established chronic inflammatory disease contributed to intraocular calcification and ossification. Here, we report a case of osseous metaplasia incidentally found as a large solid mass during the vitreoretinal surgery from a patient diagnosed with advanced Coat’s-like retinopathy and chronic retinal detachment. Previous case reports on intraocular osseous metaplasia often presented as calcified plaques rather than a solid mass. Additionally, we conducted immuno-histochemical staining to acquire more information on the pathogenic hypothesis in intraocular ossification.

## Case presentation

**Fig. 1:**
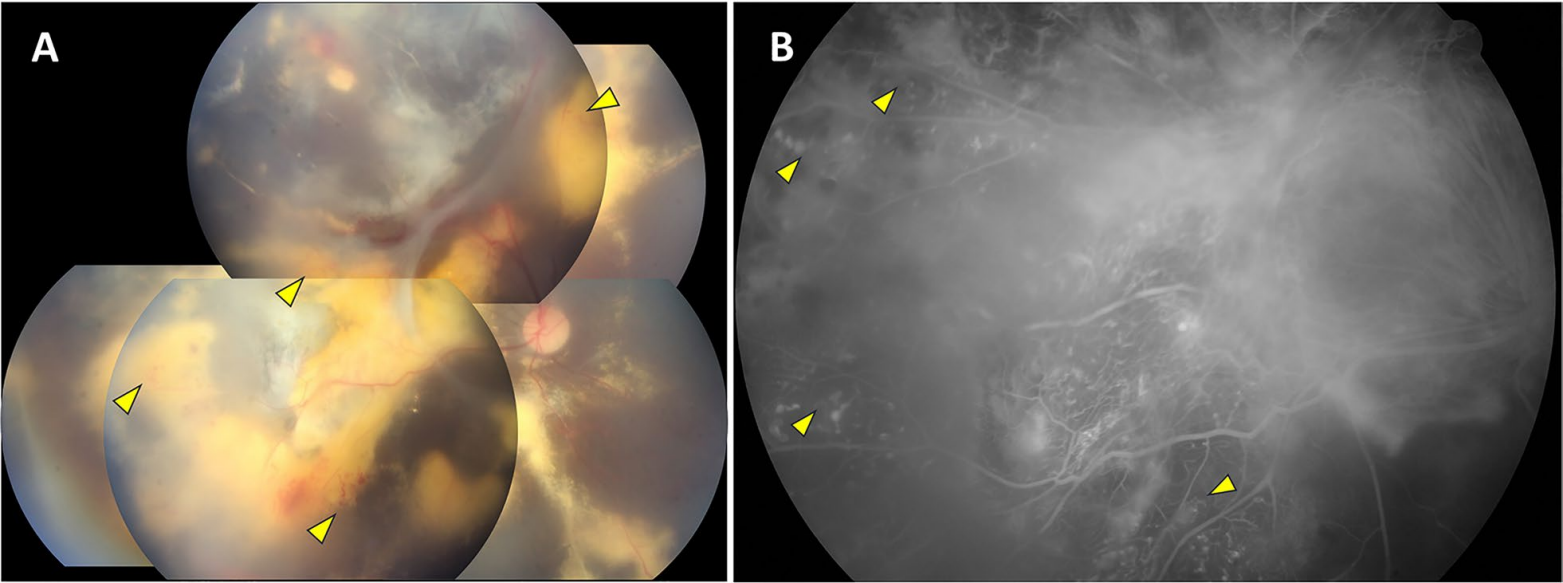
**A** Color fundus photography in the right eye shows extensive lipid depositions, exudative retinal detachment at temporal upper retina, and telangiectasic vessels in the periphery (arrowheads). **B** The fluorescein angiographic showed diffuse fluorescein dye leakage and multiple telangiectasias (arrowheads)

A 48-year-old female came to Dr. Chou’s clinic with a long-standing visual loss in her right eye for 12 years, earlier diagnosed with panuveitis in another medical unit. She denied any ocular surgery or trauma before. According to the electronic medical records from her earliest visits in our hospital 12 years ago, her vision was counting fingers in the right eye and 20/20 in the left eye. Anterior segment examination of the right eye showed iris posterior synechiae; dilated fundoscopic examination revealed diffuse lipid deposition, exudative retinal detachment, and multiple telangiectasias (Fig. 1A). No retinal holes were identified. Fluorescein angiography findings were compatible with Coat’s-like retinopathy (Fig. 1B). The left eye examination was unremarkable. She had been treated with multiple intravitreal injections and laser photocoagulation by her previous doctor. Given that little vision improvement from the medical treatment, the patient had refused any treatment since eight years ago except cataract surgery in her right eye. She had been followed up irregularly at our clinic since then.

**Fig. 2:**
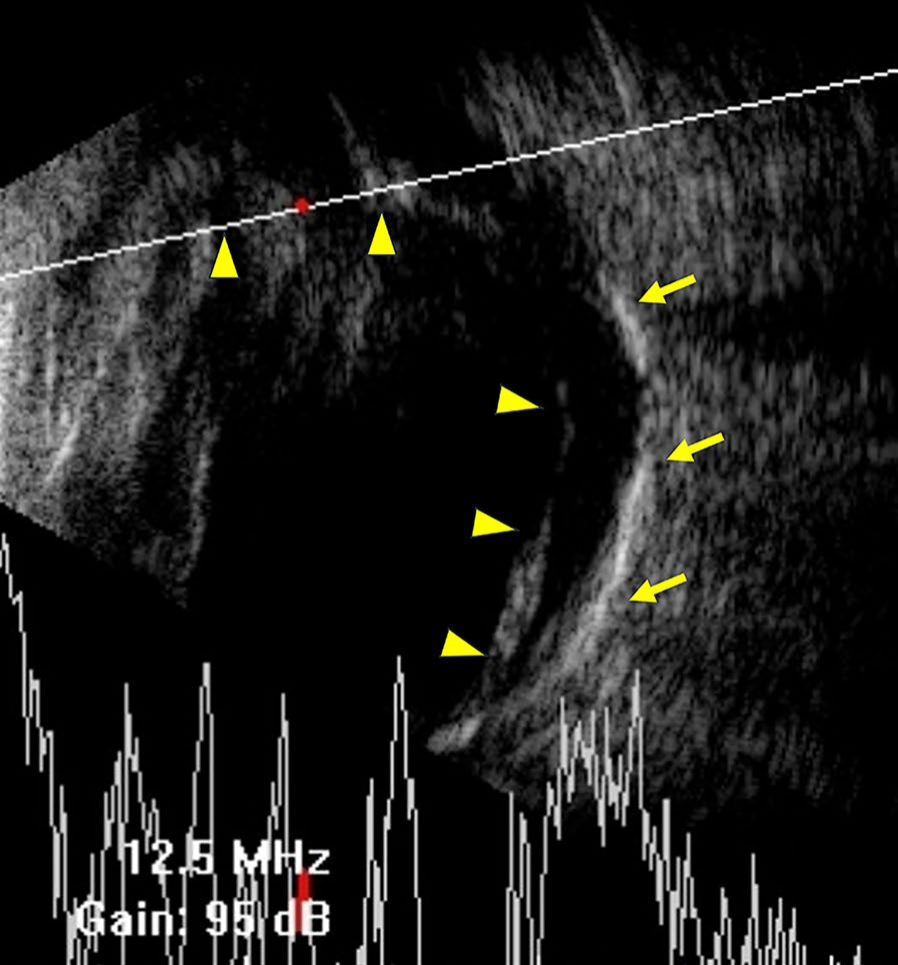
B-scan ultrasonography reveals total retinal detachment (arrowheads) and hyperreflective linear foci (arrows) consistent with intraretinal calcification that is compatible with chronic retinal detachment

This time, in September 2020, she sought medical help from Dr. Chou due to the complaint about the intolerable worsening of vision loss spanning 2 years. On examination, the best-corrected visual acuity dropped to light perception in the right eye. Anterior segment examination revealed dense fibrotic membrane covering on the intraocular lens. The fundoscopic view was totally blocked by the extensive proliferative membrane. Ultrasonography showed total retinal detachment (Fig. 2). Diagnosed with chronic retinal detachment and severe proliferative vitreoretinopathy secondary to Coat’s-like retinopathy, she agreed on vitreoretinal surgery to prevent phthisis bulbi in the future.

**Fig. 3:**
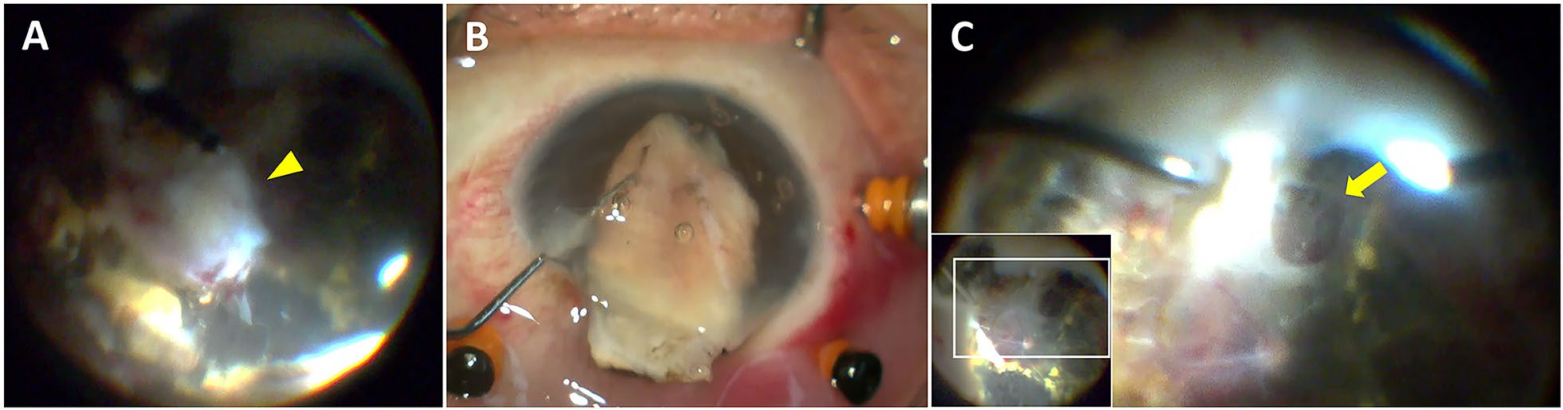
**A** A solid intraocular ellipsoid mass with a size of 6 mm in length, 4 mm in width, and 9 mm in height was incidentally found at the temporal lower area of the anterior retina during the vitreoretinal surgery (arrowhead). It was embedded in the preretinal proliferative fibrotic tissue, **B** and was removed from the anterior chamber with the enlargement of the corneal wound after the intraocular lens and capsular bag were removed beforehand. **C** A large retina break was seen at the superior retina after removing proliferative fibrotic tissue within the vitreous cavity (arrow)

**Fig. 4:**
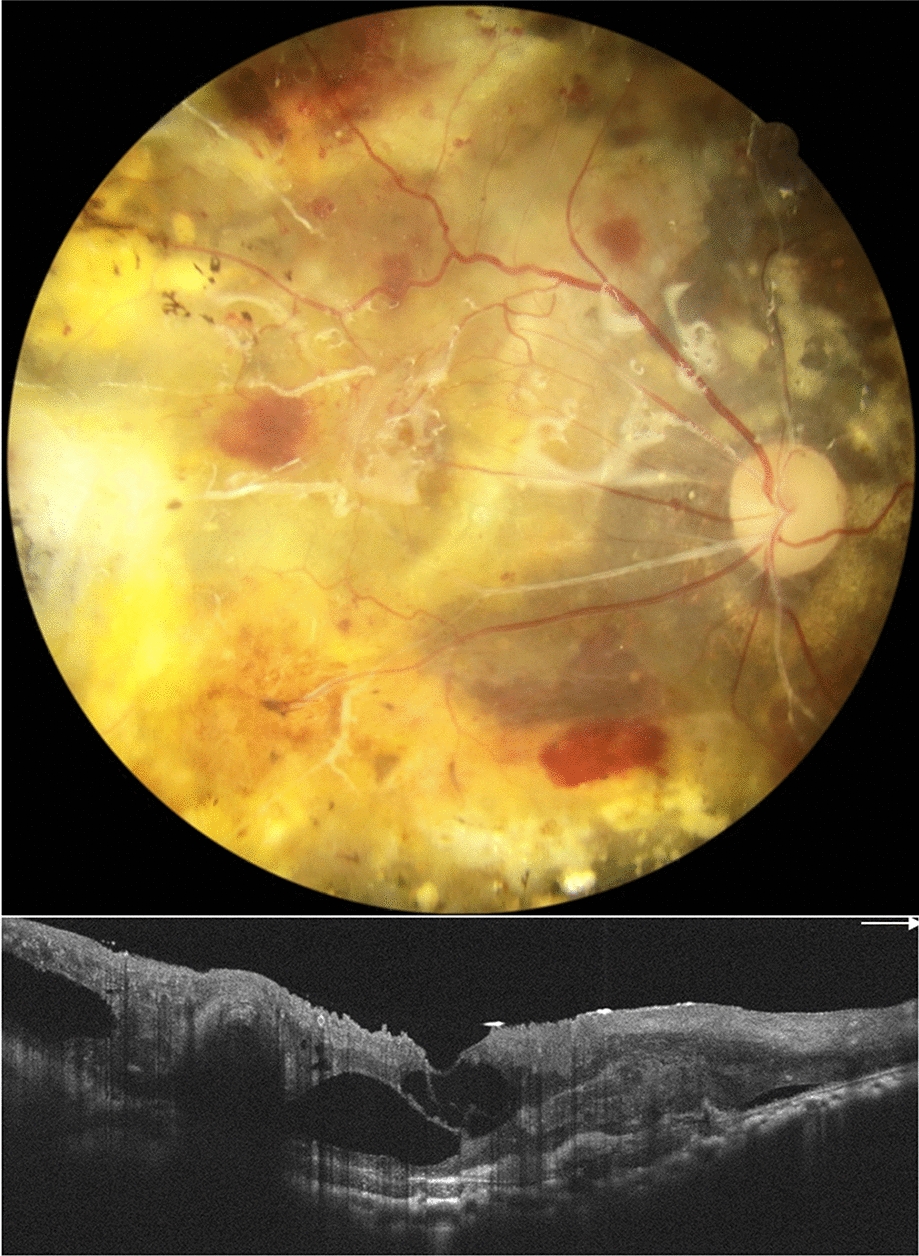
Postoperative fundus photography (upper figure) and optical coherence tomography (lower figure) of the right eye showed attached retina with intraretinal cysts

During the surgery, an incidental finding of a huge ellipsoid mass incorporated with preretinal fibrotic tissue at the anterior portion posterior to the ora serrata was noted (Fig. 3A), after the removal of anterior proliferative membrane by dissection with intraocular scissors (Fig. 3B). This intraocular mass was found so stony hard that the vitrector was unable to cut it into pieces. Hence, the tumor-like mass was extensively dissected by intraocular scissor and vitrector alternatively. It was removed from the anterior chamber by a foreign-body forcep through the corneal wound, while beforehand the intraocular lens was explanted along with the capsular bag without a secondary implant. The intraocular mass was sent for pathological analysis. Also, a large retinal break superior to the disc was seen intraoperatively following the removal of posterior fibrotic membrane (Fig. 3C). The surgery was completed with vitrectomy, membrane dissection, drainage of subretinal fluid, endolaser, and silicone oil injection. Postoperatively, the retina was attached with intraretinal cysts (Fig. 4). The final visual acuity of her right eye at the 6-month follow-up was stabilized at light perception, and intraocular pressure was 14 mmHg.

## Pathological findings

**Fig. 5:**
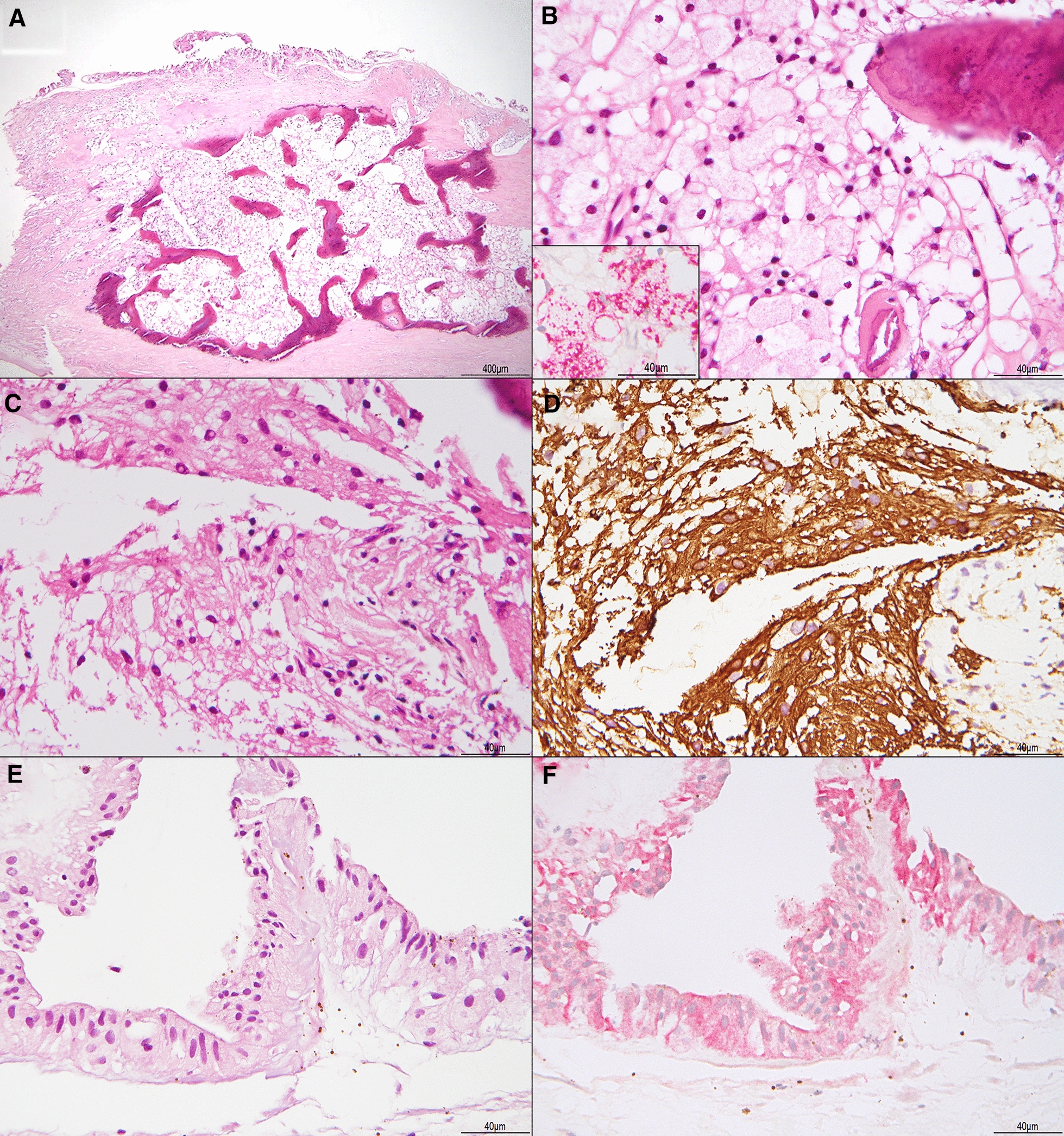
The tumor-like mass reveals osseous metaplasia. **A** A large focus of metaplastic bone containing marrow-like tissue was present within the fibrotic mass (H&E stain, 40  ×  magnification). **B** Osteoblasts-rimming woven bones and abundant lipid-laden macrophages are identified in the marrow-like tissue without cellular atypia (H&E stain, 400  ×  magnification; inset: CD68 stain, 400  ×  magnification). **C** Surrounding the fibro-osseous tissue is gliotic tissue (H&E stain, 400  ×  magnification) **D** that is highlighted with glial fibrillary acid protein (GFAP) stain. **E** A monolayer to several layers of cohesive cells lining along the fibro-osseous tissue were identified. These cells displayed cuboidal to columnar shape with some melanin pigments (H&E stain, 400  ×  magnification). **F** Immunohistochemically they are positive for CK8 (CAM5.2) and negative for CD68 (not shown), suggesting a retinal pigment epithelial (RPE) origin (400  ×  magnification)

The surgical specimen was a grey-white hard ellipsoid mass measuring 6 mm in length, 4 mm in width, and 9 mm in height. The histopathological examination was performed by routine light microscopy with hematoxylin-eosin-stained sections showing histological features of osseous metaplasia, in which an area of marrow-like tissue with woven bone formation was shown in the center (Fig. 5A, B). Next to the osseous metaplasia, massive fibrotic tissue and focal gliotic changes were found to encapsulate the osseous center (Fig. 5C, D). We also discovered layers of cohesive cells with a few melanin pigments on the external side, lining adjacently to the fibrous metaplasia (Fig. 5E). These cohesive, epithelial-like cells are positive for CK8 (CAM5.2) (Fig. 5F) and negative for CD68. CK8 (CAM5.2) has been demonstrated to be expressed in normal and reactive human retinal pigment epithelium (RPE). Along with the negative expression of CD68, the immunohistochemical findings suggest that these cells are not melanin-laden histiocytes, but RPE cells origin instead. There is no remaining retina tissue identified in the sections.

## Discussion and conclusions

Intraocular osseous metaplasia, though rare, has been commonly reported in retinal detachment, uveitis, and ocular trauma [1]. Based on our patient’s ophthalmic history, chronological examinations, and surgical findings, we presume that the etiology of osseous metaplasia in our case is combined with chronic inflammation and chronic retinal detachment. Histological findings from our case correspond to the previously proposed pathogenesis that the formation of intraocular osseous metaplasia originates from RPE hyperplasia [4]. In current understanding, RPE cells are known to be multipotent, in which they have the ability to transform and differentiate into mesenchymal cells, such as fibroblasts and osteoclasts, with a proper inflammatory stimulus from the cytokines released from RPE cells [4, 5]. RPE hyperplasia in our patient is suggested by the morphology and immunoprofile of the multi-layered pigmented cohesive cells. We suggest that the organized lining of RPE cells, fibrotic elements, and ossified tissue represent the epithelial-mesenchymal transformation of RPE cells into osteoprogenitor cells, eventually contributing to ectopic ossification process.

Although previous studies mostly reported that RPE hyperplasia and ossification take place at subretinal or intraretinal level [5, 6], no retinal tissue but gliosis was found in our specimen. In other words, the ossification process might take place above the retina in company with proliferative vitreoretinopathy change. Previous reports believed that RPE cell migration is an essential prerequisite of preretinal ossification [7, 8]. Thus, we postulated that it was the unnoticed tractional retinal tear that occurred years after thickened proliferative tissue formed, in which RPE cells had the chance to migrate from subretinal space into the vitreous cavity leading to subsequent osseous metaplasia. Meanwhile, the retinal break exaggerated the severity of retinal detachment, which in turn enhanced the RPE transformation.

The occurrence of intraocular osseous metaplasia is believed to take more than 10 years, and it requires 20–41 years to develop extensive bony tissue [9–11]. However, in our case, the patient developed substantial intraocular ossification in a much shorter period, about 12 years. The extent of the ossification was extraordinary, in which a grossly observable tumor-like bony mass was seen, rather than linear calcifications or white flat plaques reported in previous cases [7, 12]. Given that inflammation plays a stimulating role in the osseous metaplasia process, we suggest that in our case, the inflammatory environment was more intensely built up, leading to a faster and more severe ossification process. In the early disease course of this patient, chronic uveitis and retinal detachment, acting as Coat’s-like retinopathy, drove the process gradually into osseous metaplasia in the first place. Subsequently, the presence of proliferative vitreoretinopathy accelerated the process. Accordingly, we suggest that the dual stimulus, chronic inflammation and chronic retinal detachment, contributed to the development of an ossified mass.

Although the ossified mass was indeed large, we had not been able to identified it preoperatively on ultrasonography, making us face with a structure of such dimension during the intraoperative period. Preoperatively, we had detected intraretinal calcification at the posterior pole, but extremely high amplitude signals had not been shown at the anterior portion on ultrasonography (Fig. 2). We speculated that since the lesion was located at the superior temporal part of the anterior retina close to the ora serrata, it could have been easily missed by the technician if the ultrasound probe had not been correctly oriented or properly tilted. To improve visualization of any lesion in the anterior retina, we could have instructed the patient to slightly rotate their eyes by looking at different directions, providing better cross-sectional views of the anterior retina in preoperative evaluation. Also, given that the substantial amount of proliferative vitreoretinaopathy also led to hyperreflective signals on ultrasonography, it might have interfered with the identification and measurements of calcified areas. Hence, in such severe calcified cases, computed tomography (CT) could have provide another option for preoperative survey.

In conclusion, we report a case of intraocular osseous metaplasia presented as a tumor-like mass in a middle-aged woman on the basis of advanced Coat’s-like retinopathy and chronic retinal detachment. The tumor-like mass was embedded within the proliferative fibrous tissue. In pathological investigation, immuno-histochemical results provided more evidence on the pathological process of osseous metaplasia, which includes RPE migration, hyperplasia, and transformation. It is rare for intraocular ossification appears as a large mass. Hence, we postulate that the vigorous, long-standing inflammatory environment in this patient led to a shorter process and greater extent of ossification. Additionally, it reminds us that benign cellular metaplasia could be one of the differential diagnoses of an intraocular unknown mass, especially in a situation of chronic inflammation and retinal detachment.

## Data Availability

Not applicable.

## Abbreviations

RPE: Retinal pigment epithelium

## References

[1] Finkelstein EM, Boniuk M (1969). Intraocular ossification and hematopoiesis. Am J Ophthalmol.

[2] Schnaudigel OE (1989). Intraocular ossification. Klin Monbl Augenheilkd.

[3] Vemuganti GK, Honavar SG, Jalali S (2002). Intraocular osseous metaplasia. A clinico-pathological study. Indian J Ophthalmol.

[4] Toyran S, Lin AY, Edward DP (2005). Expression of growth differentiation factor-5 and bone morphogenic protein-7 in intraocular osseous metaplasia. Br J Ophthalmol.

[5] Jakobiec FA, Thanos A, Stagner AM, Grossniklaus HE, Proia AD (2016). So-called massive retinal gliosis: a critical review and reappraisal. Surv Ophthalmol.

[6] Miller DM, Benz MS, Murray TG, Dubovy SR (2004). Intraretinal calcification and osseous metaplasia in coats disease. Arch Ophthalmol.

[7] Loewenstein JI, Hogan RN, Jakobiec FA (1997). Osseous metaplasia in a preretinal membrane. Arch Ophthalmol.

[8] Shah V, Vemuganti GK, Jalali S, Das T (2002). Osseous metaplasia in an epiretinal membrane in Eales disease. Retina.

[9] Wang Y, Cui W, Liu R, Tian Y, Ni W, Zhou C (2020). Silicone oil-associated extensive intraocular ossification: a case report. Eur J Ophthalmol.

[10] Lawson BM, Reddy SG, Jody NM (2018). Extensive intraocular osseous metaplasia with bone marrow formation. JAMA Ophthalmol.

[11] Ekinci Koktekir B, Karabagli P, Gonul S, Bozkurt B, Gedik S (2014). Extensive bone formation in a painful blind eye. J Craniofac Surg.

[12] Yoon YD, Aaberg TMS, Wojno TH, Grossniklaus HE (1998). Osseous metaplasia in proliferative vitreoretinopathy. Am J Ophthalmol.

